# Single-Dose Pharmacokinetics of Fentanyl Buccal Soluble Film

**DOI:** 10.1111/j.1526-4637.2010.00875.x

**Published:** 2010-07

**Authors:** Niraj Vasisht, Larry N Gever, Ignacio Tagarro, Andrew L Finn

**Affiliations:** * BioDelivery Sciences International, Inc.Raleigh, North Carolina; † Meda Pharmaceuticals, Inc.Somerset, New Jersey, USA; ‡ Meda Pharma S.A.U.Madrid, Spain

**Keywords:** Breakthrough Pain, Buccal Absorption, Buccal Soluble Film, Drug Absorption, Fentanyl, Pharmacokinetics

## Abstract

**Objective:**

The objectives of the study were to determine the absolute bioavailability of fentanyl from fentanyl buccal soluble film, estimate the percentage of a fentanyl dose absorbed through the buccal mucosa, and compare the bioavailability of equivalent doses administered either as single or multiple dose units.

**Design:**

Open-label, randomized, four-period, Latin-square crossover pharmacokinetic study.

**Setting:**

Inpatient phase 1 unit.

**Patients:**

Twelve healthy volunteers.

**Interventions:**

Injectable fentanyl citrate (200 µg) administered by intravenous infusion, injectable fentanyl citrate (800 µg/16 mL) administered orally, and fentanyl buccal soluble film (800 µg) administered as a single film and as four separate 200 µg films simultaneously.

**Outcome Measures:**

Plasma concentrations after fentanyl dosing; pharmacokinetic parameters.

**Results:**

The two buccal film treatments were bioequivalent and both had an absolute bioavailability of 71%. The percentage of an administered dose absorbed through the buccal mucosa was calculated to be 51%.

**Conclusions:**

Fentanyl buccal soluble film effectively delivers a high percentage of the administered fentanyl dose and nearly identical plasma profiles are obtained when equivalent doses are delivered by single or multiple dosage units.

## Introduction

Breakthrough pain has been estimated to occur in approximately 65% of patients with cancer whose pain is significant enough to require opioid analgesics [1]. Management of breakthrough pain in patients with cancer is a challenge because onset of breakthrough pain is frequently unpredictable, and episodes vary in both intensity and duration [2]. Breakthrough pain episodes can be associated with the end of a dosing interval in patients with inadequate baseline analgesia, may be associated with a precipitating event, or may be spontaneous. Episodes can reach maximal intensity within 3–5 minutes and occur with a mean frequency of four to seven times daily [3,4]. Immediate-release oral opioids are frequently used for the treatment of breakthrough pain, but analgesia may be delayed within the required therapeutic time frame [2].

The ideal drug for breakthrough pain would produce analgesia in a time frame temporal to the pain episode. However, this is only achievable with patient-controlled intravenous analgesia [2]. Alternative routes of administration differ significantly from this ideal in speed of onset, consistency of effect, and patient convenience. The oral route is commonly selected for convenience, but analgesia may be delayed due to the time required to reach intestinal absorption sites as well as by delays in gastrointestinal motility associated with a background opioid, or reduced by first-pass metabolism. Additionally, oral delivery of analgesics can be problematic for patients who have difficulty swallowing, are nauseated, or have other gastrointestinal conditions.

Alternative dosage forms that provide acute relief of breakthrough pain may be beneficial for appropriate patients.

Buccal administration can deliver lipophilic opioids rapidly to the systemic circulation through the buccal mucosa, limiting gastrointestinal motility and first-pass metabolism [5]. Two forms of fentanyl citrate have been approved in the United States for the treatment of breakthrough pain in patients with cancer including an oral transmucosal fentanyl citrate (OTFC) lozenge (Actiq®, Cephalon, Inc., Frazer, PA) and a fentanyl effervescent buccal tablet (FBT) (US: Fentora®, Europe: Effentora®, Cephalon, Inc.; Frazer) [6]. A relatively wide range of variability in plasma concentrations has been reported with the OTFC lozenge, likely because the fraction of the dose that is swallowed may vary from dose to dose depending upon how exactly the device is applied at each occasion. With FBT [7], the dosing range is limited by nonlinear pharmacokinetics above 800 µg [8]. Further, there are differences in pharmacokinetics between multiple units of a lower strength (4 × 100 µg) and a single higher strength (400 µg) that authors attributed to differences in the absorptive surface area [9].

A mucoadhesive, buccal-soluble film (Meda Pharmaceuticals, Inc., Somerset, NJ) has also been approved in the United States using the BioErodible MucoAdhesive (BEMA™, BioDelivery Sciences, Inc., Raleigh, NC) delivery technology. Once applied to the oral mucosa, the fentanyl buccal soluble film (FBSF) (US: Onsolis®, Meda Pharmaceuticals Inc.) adheres to the mucosa in seconds. Fentanyl is contained in the mucoadhesive layer, and a second inactive layer separates the fentanyl-containing layer from the saliva and limits the amount of fentanyl that is swallowed. Each film is composed of water-soluble polymers that completely dissolve so there is no residual product to be removed, and there is a direct proportional relationship between the surface area of the film and the administered dose.

The objectives of this open-label, randomized, single-dose, crossover pharmacokinetic study were to determine the absolute bioavailability of fentanyl from this advanced transmucosal delivery system, estimate the percentage of a fentanyl dose that is absorbed through the buccal mucosa, and compare the bioavailability of equivalent doses administered either as single or multiple dose units.

## Methods

This was an open-label, randomized, four-period, Latin-square crossover study. The study protocol was approved by a regional institutional review board, and the study was conducted in accord with the principles of the Declaration of Helsinki and the US Code of Federal Regulations (Title 21, Part 50). All subjects read and signed an approved informed consent form during screening.

### Participants

Subjects eligible for inclusion in the study were healthy adult male and nonpregnant, nonlactating female volunteers aged 18–45 years, weighing 60–100 kg, and within 15% of their ideal body weight based on Metropolitan Life tables for height and weight. Premenopausal women were required to use an acceptable method of birth control and to have a negative urine test for β-human chorionic gonadotropin. Consumption of alcoholic beverages, caffeine- or xanthine-containing beverages, or foods or beverages containing grapefruit was prohibited from 48 hours prior to the first dose of study medication until discharge from the study. Use of tobacco or nicotine-containing products within 30 days of the first dose of study medication was also not allowed.

Subjects were excluded if they had participated in an investigational drug study within the previous 30 days; had taken any nutritional supplement or any prescription or nonprescription medication (except acetaminophen or oral contraceptives) within 72 hours of the first dose of study medication; or had a positive drug screen for amphetamines, barbiturates, benzodiazepines, cannabinoids, cocaine or opiates, or a positive ethanol breath test. A history of a serious medical condition, including glaucoma or a seizure disorder, and allergy or intolerance to narcotics were also grounds for exclusion.

### Trial Design and Procedures

The subjects were assessed at a screening visit and confined to a phase 1 unit throughout the 12-day treatment period. The Latin-square crossover study design was used to minimize variability by ensuring each subject received a single dose for each treatment each period. Each subject received the following four study treatments on days 1, 4, 7, and 10: injectable fentanyl citrate 200 µg administered via an intravenous infusion pump over 5 minutes; injectable fentanyl citrate 800 µg/16 mL administered orally via a 20 mL oral syringe followed by 90 mL of water; fentanyl 800 µg as a single film applied to the buccal mucosa; and fentanyl 800 µg administered as four separate 200 µg films applied to the buccal mucosa within a 2-minute period. The 800 µg film provided a surface area that is four times larger than that of the 200 µg one (3.1 cm^2^ compared with 0.78 cm^2^). Each drug administration was separated by a washout period of at least 72 hours.

A commercially available injectable fentanyl citrate product (Fentanyl Citrate Injection, Hospira, Inc., Lake Forest, IL; Lot 44-296-DK, Expiration August 1, 2008) was used for the intravenous and oral doses. The buccal dosage form contained fentanyl citrate, but the dosage is expressed as fentanyl free base.

Prior to application of the buccal soluble film doses, the subjects rinsed their mouths with water. Buccal doses were placed on the mucosa inside the cheek below the level of the lower teeth. Each film was then applied to the mucosa and held in place for a few seconds until it was moistened by saliva and adhered to the mucosal membrane. The subjects were instructed not to rub the film with their tongue. With the four-film regimen (D), two films were applied to the posterior portion and two to the anterior portion of the contralateral sides of the mouth.

All of the subjects received oral naltrexone 50 mg approximately 12 hours and 1 hour prior to, and approximately 12 hours after study drug administration to block the respiratory depressive effects of fentanyl in these opioid-naïve subjects. Predose procedures were performed on the evening of day 0 and the subjects received the study drug approximately 1 hour after consuming a standard light breakfast on days 1, 4, 7, and 10. The subjects fasted for 4 hours after receipt of study medication.

Blood samples for measurement of fentanyl plasma concentrations were collected prior to drug administration (0 hour) and at 5, 10, 15, 20, 30, 45, and 60 minutes, and 2, 3, 4, 8, 12, 16, 20, 24, and 48 hours after each dose of the study drug. Pharmacokinetic sampling times began with the application of the first film when the subjects received the four-film regimen.

### Sample Collection and Pharmacokinetic Analysis

Venous blood samples (7 mL) were collected in EDTA Vacutainer® tubes (BD, Franklin Lakes, NJ). Within 30 minutes of collection, the samples were centrifuged and the plasma fraction removed and stored at −20°C pending analysis.

Fentanyl concentrations in plasma were determined by a validated liquid chromatography with tandem mass spectrometry method that has a lower limit of quantification of 0.0250 ng/mL and an upper limit of quantification of 5.00 ng/mL. Fentanyl-D_5_ was used as the internal standard. Quantification was performed by a weighted (1/X^2^) linear least squares regression analysis that was generated from calibration standards. Bioequivalence between the 4 × 200 µg and 1 × 800 µg FBSF was determined by comparison of the 90% confidence intervals (CIs) for the log-transformed exposure parameters C_max_, AUC_last_, and AUC_inf_ against the accepted 80% to 125% range.

The following pharmacokinetic parameters were calculated: C_first_ = first measurable drug concentration in plasma determined directly from individual concentration time data; C_max_ = maximum drug concentration in plasma determined directly from individual concentration time data; T_max_ = time to C_max_; λ_z_ = observed elimination rate constant estimated by linear regression through at least three points in the terminal phase of the log concentration time profile for each subject; t_1/2_ = observed terminal elimination half-life calculated as ln(2)/λ_z_; AUC_0–48_ = area under the drug concentration time curve from time 0–48 hours calculated using the linear trapezoidal rule and extrapolated using λ_z_ if measurable plasma concentrations were not obtained throughout the 48-hour sampling period; AUC_last_ = area under the drug concentration time curve calculated using linear trapezoidal summation from time zero to the time of the last measurable concentration; AUC_inf_ = area under the drug concentration time curve from time zero extrapolated to infinity calculated as AUC_last_ + C_last_/λ_z_.

The absolute bioavailability of fentanyl was determined using the following equation:


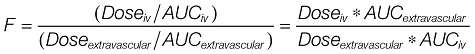


*Dose_extravascular_* and *AUC_extravascular_* are those that pertain to oral or buccal administration. Mean AUC_inf_ was used in these calculations. The percentage of the dose absorbed through the buccal mucosa was estimated by subtracting the AUC_inf_ after oral administration from that after buccal administration, dividing by the AUC_inf_ after buccal administration, and multiplying by 100.

### Safety Assessments

Safety monitoring was composed of physical examination, vital signs, 12-lead electrocardiogram, laboratory studies, and evaluation of subjects for adverse events and monitoring of oxyhemoglobin with a pulse oximeter.

### Statistics

Plasma concentration time data were analyzed by noncompartmental methods using WinNonlin® (Pharsight Corporation, Mountain View, CA). Pharmacokinetic calculations were based on actual sampling times. The statistical analysis was performed using SAS version 8.2 (SAS Institute Inc., Cary, NC).

Plasma fentanyl concentrations that were below the limit of quantification after drug administration were assigned a value of zero if collected prior to C_max_ and were treated as missing values if collected after C_max_. For the subjects with measurable concentrations in predose samples, the first measurable concentration above the predose level after drug administration was redefined as the first measurable concentration.

The sample size chosen for this study was based on conventional pharmacokinetic study designs, not a formal power calculation.

Natural log-transformed values for C_max_, AUC_last_, and AUC_inf_ were analyzed for differences between treatments and gender using an analysis of variance (anova) model and Schuirmann's two one-sided *t*-test procedures at the 5% significance level, analogous to the analysis used for bioequivalence assessments; the ratio of the geometric means and the 90% CIs were reported [10].

Values for T_max_ after administration of a single 800 µg buccal film or four individual 200 µg films were compared with the Wilcoxon signed-rank test with a significant difference defined as *P* < 0.05.

## Results

A total of 12 subjects were enrolled, each of whom completed all four treatment periods. A summary of the demographic characteristics of the participants in the study is contained in Table 1. The mean age of the subjects was 27 years, 50% were men, and 50% were black. Measurable plasma concentrations of fentanyl were detected in predose (0 hour) samples collected for some of the subjects during periods 2, 3, and 4. The concentration of fentanyl in all such samples was <5% of the C_max_ for the profile in question, and as such, the concentrations were included in the analysis without adjustment.

**Table 1:** Demographic characteristics

Number of volunteers	12
Male : female gender	6:6
Mean age in years ± SD (range)	27 ± 6 (19–37)
Race, n (%)	
Black	6 (50)
Caucasian	4 (33)
Hispanic or Latino	2 (17)
Mean height in cm ± SD (range)	169.0 ± 9.0 (154.5–180.5)
Mean weight in kg ± SD (range)	70.7 ± 6.4 (63.0–84.1)

SD = standard deviation.

### Single-Dose Pharmacokinetics

**Figure 1:**
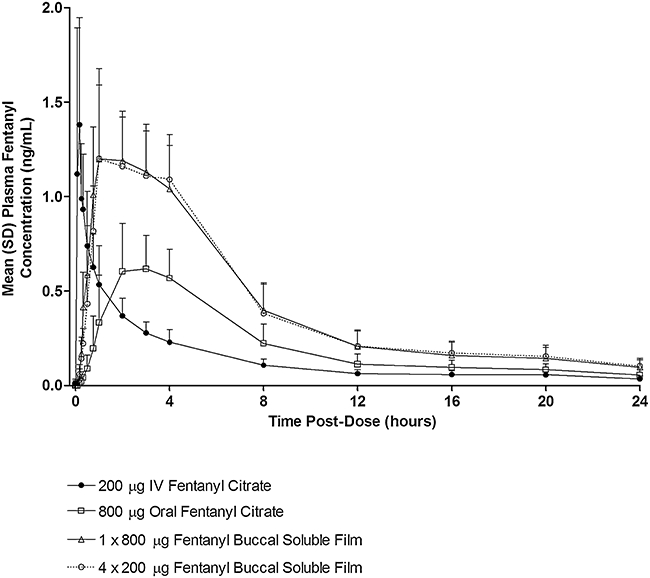
Mean plasma concentration profile. IV = intravenous; SD = standard deviation.

The plasma concentration time profile of fentanyl administered by the intravenous, oral, and buccal routes is illustrated in Figure 1. The profiles for the two buccal treatments overlap very closely and are distinct from those for oral and intravenous administration.

Pharmacokinetic parameters for fentanyl in each of the treatment periods are presented in Table 2. The mean C_max_ of fentanyl was identical (1.33 ng/mL), and exposure as measured by mean AUC_inf_, was nearly identical (13.03 vs 13.09 hours/ng/mL) after administration of the two buccal treatments (Table 2). Following the buccal doses, mean C_max_ and AUC_inf_ were 1.9 and 2.0 times that of oral administration. The absolute bioavailability of fentanyl from the buccal soluble film treatments was 71%, which is approximately double that after oral administration (35%). The percentage of an administered dose absorbed through the buccal mucosa was calculated to be 51%.

**Table 2:** Single-dose pharmacokinetics of fentanyl after intravenous, oral, and buccal administration

Parameter	A: IV Administration (200 µg Fentanyl Citrate)	B: Oral Administration (800 µg Fentanyl Citrate)	C: Buccal (1 × 800 µg Film)	D: Buccal (4 × 200 µg Films)
Mean C_max_ ± SD, ng/mL (CV%)	1.46 ± 0.66 (44.97)	0.69 ± 0.21 (30.21)	1.33 ± 0.31 (23.01)	1.33 ± 0.43 (32.30)
Mean AUC_last_ ± SD, hour/ng/mL (CV%)	3.78 ± 1.18 (31.16)	5.65 ± 2.09 (37.01)	11.40 ± 3.03 (26.57)	11.70 ± 3.20 (27.37)
Mean AUC_inf_ ± SD, hour/ng/mL (CV%)	4.62 ± 1.51 (32.76)	6.39 ± 2.28 (35.63)	13.03 ± 3.45 (26.50)	13.09 ± 3.62 (27.62)
Mean AUC_extrap_ ± SD, (CV%)	17.75 ± 5.44 (30.63)	11.84 ± 3.16 (26.66)	12.23 ± 6.77 (55.35)	10.57 ± 3.37 (31.91)
Mean λ_z_ ± SD, hours^−1^ (CV%)	0.052 ± 0.032 (60.96)	0.061 ± 0.023 (37.91)	0.042 ± 0.016 (37.32)	0.04 ± 0.01 (26.71)
Median T_max_; range, hours	0.17; 0.08–0.37	3.00; 1.00–4.00	1.50; 0.75–4.00	2.50; 1.00–4.00
Mean t_1/2_ ± SD, hours (CV%)	18.03 ± 10.08 (55.91)	13.26 ± 5.68 (42.80)	19.03 ± 8.31 (43.67)	18.29 ± 4.14 (22.61)

Values are presented as arithmetic means ± SD.

AUC = area under the drug concentration time curve; C_max_ = maximum observed plasma drug concentration; CV = coefficient of variation; SD = standard deviation; T_max_ = time to maximum plasma drug concentration; t_1/2_ = elimination half-life; λ_z_ = elimination rate constant.

Pharmacokinetic parameters were generally similar in the males and the females after administration of a single 800 µg buccal dose of fentanyl (Table 3). The exception was the mean t_1/2_, which was longer in the females (15.6 hours) than in the males (10.9 hours), a finding that may be attributable to particularly long half-lives in two individuals. When analyzed by anova, the female : male ratios of the geometric mean values for C_max_, AUC_last_, and AUC_inf_ were 94.46%, 93.89%, and 102.5%, respectively. Due to the small sample size (i.e., six female and six male subjects), the power associated with these comparisons is low (0.44, 0.34, and 0.34, respectively).

**Table 3:** Statistical analysis of fentanyl plasma pharmacokinetic parameters in female and male subjects after administration of a single 800 µg fentanyl buccal soluble film

	Geometric LSM*		
Parameter	Female	Male	Ratio of LSM for Female/Male, %
ln C_max_	1.265	1.337	94.46
ln AUC_last_	10.693	11.389	93.89
ln AUC_inf_	12.738	12.483	102.05

* Values are the least squares means (LSMs). LSMs are the average of means associated with a treatment.

AUC_inf_ = area under the drug concentration time curve from time zero extrapolated to infinity; AUC_last_ = area under the drug concentration time curve from time zero to the time of the last measurable concentration; C_max_ = maximum observed plasma drug concentration.

There were no differences in bioavailability in terms of either the rate or extent of absorption of fentanyl after administration by the buccal route as a single 800 µg film or as four 200 µg films (Table 4). The median T_max_ occurred later after administration of fentanyl as four 200 µg films compared with a single 800 µg film (2.5 vs 1.5 hours, respectively), although the difference was not statistically significant (*P* = 0.0781 by Wilcoxon signed-rank test).

**Table 4:** Exposure to fentanyl after administration of a single 800 µg fentanyl buccal soluble film or four 200 µg fentanyl buccal soluble films

	Geometric LSM*			
Parameter	Four × 200 µg Films	Single 800 µg Film	Ratio of LSM for 4 × 200 µg/800 µg, %; 90% CI	anova CV%
ln C_max_	1.269	1.301	97.50; 90.50–105.04	9.83
ln AUC_last_	11.283	11.036	102.24; 96.61–108.21	7.48
ln AUC_inf_	12.625	12.610	100.12; 94.00–106.64	8.32

* Values are the least squares means (LSM).

anova = analysis of variance; AUC_inf_ = area under the drug concentration time curve from time zero extrapolated to infinity; AUC_last_ = area under the drug concentration time curve from time zero to the time of the last measurable concentration; CI = confidence interval; C_max_ = maximum observed plasma drug concentration; CV = coefficient of variation.

anova CV% is the square root of the residual variance times 100.

### Safety

No serious adverse events were reported during the study and no subject withdrew from treatment because of adverse events. A total of nine adverse events were reported by three subjects. All adverse events were considered to be mild in intensity and all were resolved spontaneously. Contact dermatitis and menstrual cramps were reported after intravenous administration (N = 1 each); loose stool, nausea, and skin rash were reported after oral administration (N = 1 each); dizziness and headache were reported after buccal administration of a single 800 µg buccal film (N = 1 each), and constipation and headache were reported after administration of four 200 µg buccal films (N = 1 each). No clinically significant observations or changes in vital signs, physical examinations, electrocardiograms, or clinical laboratory tests were identified in the subject population during the study.

## Discussion

Based on AUC_inf_ estimates, this study showed that the bioavailability of FBSF was 71%, which was greater than the bioavailability after oral fentanyl (approximately 35%). As determined by pharmacokinetic calculation, the percentage of the fentanyl dose absorbed through the buccal mucosa was estimated to be 51%. Overall systemic exposure as assessed by AUC and C_max_ was nearly identical after administration of 1 × 800 µg and 4 × 200 µg FBSF. No differences in bioavailability were observed in the rate and extent of absorption between 1 × 800 µg and 4 × 200 µg dosages, indicating that multiple small dose units can be used interchangeably with higher dose units. Furthermore, the pharmacokinetic profile of FBSF was not gender-dependent, with similar values for both males and females for the exposure parameters of C_max_, AUC_last_, and AUC_inf_. Adverse events with FBSF were mainly gastrointestinal and central nervous system disorders that were mild in intensity and resolved spontaneously.

The safety results we report here are similar to that of other formulations of fentanyl [11]. The lack of gender effects reported here is different from the results previously reported for the fentanyl buccal tablet, which reported a 20% to 30% increased systemic exposure for females [4].

In a comparative crossover study conducted in 26 volunteers, the following absolute bioavailability ratios were reported: FBT, 65%; OTFC, 47%; and FBT administered orally, 31% [11]. Although the bioavailability reported here for FBSF (71%) is higher than that reported for FBT (65%), the clinical relevance of this cross-comparison is not known. The buccal absorption rate of 51% reported here is comparable with the 48% transmucosal absorption for FBT and more than two-fold higher than the 22% transmucosal absorption reported for OTFC [11].

FBSF as examined in this study has distinct pharmacokinetic properties in comparison with other commercially available fentanyl dosage forms intended for buccal administration. The study demonstrates that a buccal soluble film dosage form delivers fentanyl with high absolute bioavailability (71%) when administered as a single 800 µg film or as four individual 200 µg films and that the two study treatments are bioequivalent.

The high fentanyl bioavailability produced by FBSF results from the significant drug fraction that undergoes transmucosal absorption and therefore that escapes from first-pass metabolism. The effectiveness of transmucosal absorption, in turn, likely reflects the effectiveness of the film inactive layer in minimizing the amount of fentanyl that is swallowed with the saliva.

The observed bioequivalence when the same dose is administered as a single film or four different films results from the fact that the absorption produced by buccal film technology is proportional to the surface area of the film. Consequently, the absorption surface area with a single 800 µg film is exactly the same as with four individual 200 µg films.

The major limitation of the study is that it was conducted in healthy volunteers, who had to be given naltrexone to prevent respiratory depression. There may be differences in the pharmacokinetics of fentanyl as experienced by the patients with cancer, who may be undergoing chemotherapy or may be taking multiple concurrent medications. However, because the dose is set individually by titration, any such differences are not likely to be clinically relevant. Another limitation is that the washout time was at least 72 hours, which corresponds to 3.8 terminal half-lives. However, the residual concentration was <5% and was taken into account in the calculations.

In conclusion, the absolute bioavailability of fentanyl from the buccal soluble film is determined in the present study to be 71%. The direct relationship between the surface area of the film and the dose of fentanyl results in bioequivalence between a single unit and that of multiple dose units that give the same combined total dose. This aspect provides confidence in the titration of FBSF to an effective dose for the management of breakthrough pain.
